# Prevalence of relative systemic hypertension in adults with sickle cell disease in Ghana

**DOI:** 10.1371/journal.pone.0190347

**Published:** 2018-01-04

**Authors:** Amma Benneh-Akwasi Kuma, Amma Twumwa Owusu-Ansah, Mary Akua Ampomah, Fredericka Sey, Edeghonghon Olayemi, Mehdi Nouraie, Solomon Fiifi Ofori-Acquah

**Affiliations:** 1 Department of Hematology, College of Health Sciences, University of Ghana, Accra, Ghana; 2 Division of Hematology Oncology, Department of Medicine, University of Pittsburgh School of Medicine, Pittsburgh, Pennsylvania, United States of America; 3 Center for Translational and International Hematology, Vascular Medicine Institute, University of Pittsburgh, Pittsburgh, Pennsylvania, United States of America; 4 Ghana Institute of Clinical Genetics, Korle-Bu, Accra, Ghana; 5 Division of Pulmonary, Allergy and Critical Care Medicine, Department of Medicine, Heart, Lung and Vascular Medicine Institute, University of Pittsburgh School of Medicine, Pittsburgh, Pennsylvania, United States of America; Loyola University Chicago, UNITED STATES

## Abstract

Individuals with sickle cell disease particularly with the homozygous (SS) genotype historically have relatively low blood pressure. Nonetheless, they develop vasculopathy-associated organ dysfunction and the risk of organ dysfunction increases at blood pressures that are normal in the general population. This phenomenon is termed relative systemic hypertension (RSH) with a systolic blood pressure range of 120–139 mmHg, and diastolic blood pressure range of 70–89 mmHg. The significance of RSH lies in its association with renal insufficiency, pulmonary hypertension, stroke and propensity to progress to systemic hypertension. We conducted a retrospective chart review of 1,000 adults with sickle cell disease at the Ghana Institute of Clinical Genetics, to determine the prevalence of RSH in sickle cell disease in Ghana and associated complications. We found a high prevalence of RSH and hypertension with a relatively low frequency of renal insufficiency. Pulse pressure, a predictor of mortality, was higher in males of all ages. We anticipate that providing an estimate of the burden of RSH will heighten its recognition and clinical management among health care providers.

## Introduction

Sickle cell disease (SCD) is a hemoglobinopathy caused by a β-globin gene (β^s^) mutation at position 6 (Glu > Val). This mutation results in an aberrant hemoglobin (Hb) with altered physical properties, referred to as sickle hemoglobin (Hb S). Hb S polymerizes when deoxygenated, distorting erythrocytes into the characteristic sickle shape. Individuals with SCD have two copies of the β^s^ gene (SS) or one β^s^ gene in combination with hemoglobin C (SC) or β-Thalassemia (SβThal) mutations. Clinically, SCD is characterized by chronic hemolytic anemia and episodic painful vaso-occlusion of variable severity that culminates in organ dysfunction and reduced life expectancy [1, 2].

Multiple studies have shown that individuals with homozygous SCD (SS) have lower blood pressure (BP) than age, race and gender-matched controls [3–5]. Compound heterozygous states of SCD (SC or Sβ+Thal) are also associated with low BP albeit at slightly lower frequency than in SS. Anemia, hyposthenuria [4], altered vascular reactivity to angiotensin II [6], decreased peripheral vascular resistance and decreased afterload [7] are cited as probable contributors to the relatively low BP observed in SCD. However, SCD individuals have higher BP than age-matched individuals with anemia, and older males with SS with elevated BP relative to the SCD population are at increased risk of stroke [7].

Analysis of data from SS adults enrolled in the Sickle Cell Pulmonary Hypertension Screening Study identified a risk of developing, within a 2-year period, new onset or worsening pulmonary hypertension and renal insufficiency at BPs considered to be within normal range for the general population[8]. This observation birthed the term “relative systemic hypertension” (RSH), which is defined as 120–139 mmHg systolic and 70–89 mmHg diastolic BP. A parallel has been drawn between RSH and prehypertension as defined by the Joint National Committee for Prevention, Detection, Evaluation and Treatment of Hypertension (JNC), both conditions presenting a unique opportunity to institute preventive therapy to mitigate organ dysfunction from vasculopathy [8].

The birth prevalence of SCD in Ghana which has a population of approximately 28 million is 2% [9]. The prevalence of RSH in the adult SCD population in Ghana is unknown. In this study, we determined the prevalence of RSH in adults with SCD receiving care at the Ghana Institute of Clinical Genetics, Korle-Bu Teaching Hospital in the capital city Accra.

## Methods

### Study site and study population

The Ghana Institute of Clinical Genetics (GICG), Korle-Bu Teaching Hospital in Accra, Ghana has provided medical care to over 25,000 registered individuals with SCD since its inception in 1974. With over 10,000 patient visits per year, it is one of the largest SCD clinics in Africa. Patients with SCD 13 years and older are referred to GICG from other institutions or at age 16 years from the Pediatric Department of Korle-Bu Teaching Hospital.

The study population comprised adults with SCD aged ≥ 18 years who visited GICG for care at least once within the period of the study.

The Ethics and Protocol Review Committee of the School of Biomedical and Allied Health Sciences, College of Health Sciences, University of Ghana, approved the study (Ethics Identification Number: SBAHS-ET/0244365283/AA/HIA/2012-2013)

### Chart selection and data extraction

Over a 10-month period (October 2014 to July 2015), we conducted a retrospective review of 1,000 patient charts to determine the prevalence of RSH in adults with SCD receiving regular care at the GICG. The objective for our study was to provide an estimate of the prevalence of RSH in a large representative sample of adults with SCD in Ghana.

Charts were selected by convenience sampling when the patients visited the clinic for medical care. The reasons for visiting the clinic were varied and included acute illness, pain, fever, a follow-up after acute illness or a non-acute visit for comprehensive care. Chart reviews were performed by two investigators who obtained blood pressure values and dates on which they were collected, located corresponding laboratory results and averaged out laboratory variables. This data was then entered by the second investigator into a Microsoft Excel spreadsheet. Using a data extraction manual created for the study to optimize inter-rater reliability, we collected demographic, clinical and laboratory data from the charts. A unique study identification number was assigned to each chart selected. All selected charts were marked with labels bearing the study ID to avoid duplication of charts. The charts were reviewed first for eligibility as per the data extraction manual.

### Eligibility criteria

#### Inclusion criteria

Individuals 18 years or older with homozygous or compound heterozygous sickle cell disease, seen at least once at the GICG during the study period were eligible for inclusion.

#### Exclusion criteria

- Age less than 18 years
  Other non-sickle hemoglobinopathies

### Chart abstraction

From each eligible chart, the following data were collected: Hb types (as defined by hemoglobin electrophoresis), gender, age, systolic and diastolic blood pressure, dates on which blood pressures were recorded, CBC, renal function tests, presence of pre-existing diagnoses of hypertension and associated medication. Specifically, the three most recent blood pressure readings taken on three separate days were recorded. The blood pressures were obtained using an automated blood pressure monitor that had been in use in GICG for 2 years including the period of the study. We collected hemoglobin and total white blood cell (WBC) counts obtained on the same days as the blood pressures, averaged them and recorded the average values in the chart. Finally, complications attributable to SCD documented in each eligible chart were recorded.

Normal blood pressure was defined as systolic blood pressure <120mmHg and diastolic blood pressure < 70 mmHg in the absence of antihypertensive medication.

Relative systemic hypertension was defined as either a systolic blood pressure (SBP) of 120 to 139 mmHg, or a diastolic blood pressure (DBP) of 70 to 89 mmHg in a subject without documented diagnosis of hypertension and not taking antihypertensive medication.

Hypertension was defined as either blood pressure of ≥140mmHg (systolic) or ≥ 90 mmHg (diastolic) in the chart or blood pressure of any value along with documented treatment with one or more antihypertensive medications.

SCD type–The current diagnostic modality for SCD at GICG is cellulose acetate electrophoresis which can distinguish between hemoglobin S and C but is limited in distinguishing between Hemoglobin S, D and G In the absence of genetic testing or confirmatory tests such as HPLC or Isoelectric focusing to provide a definitive phenotype, the SCD phenotypes “SS, SC, SS/SD, SS/SD/SG” were assigned based on cellulose acetate electrophoresis alone. The latter two phenotypes SS/SD and SS/SD/SG reflect the limitations of cellulose acetate electrophoresis in distinguishing hemoglobin S, D, and G.

### Statistical analysis

The prevalence of RSH was calculated using single blood pressure measurements for the sample and then stratified by age, gender and genotype. The prevalence of hypertension was determined. We used ANOVA and Chi-square (or Kruskal-Wallis and Fisher’s exact test if required) to compare the continuous and categorical variables of the following three groups; those with normal blood pressure, RSH and hypertension. Multinomial logistic regression was used to detect significant predictors of RSH and hypertension. In an alternative analysis, the prevalence of RSH and hypertension among patients who had all three blood pressure readings taken within the same year was calculated using averaged systolic and diastolic blood pressure from repeated measures. A p-value of 0.05 or less was considered statistically significant. Stata 14.1 (StataCorp., College Station, TX) and GraphPad Prism version 7 were used.

## Results

Of the 1,000 charts reviewed, 875 met the eligibility criteria of having documented phenotype consistent with homozygous or compound heterozygous SCD and age ≥18 years. Of the other 125 charts, two were excluded for having non-SCD hemoglobinopathies, presumed homozygous C disease and β-Thalassemia major; the rest were aged < 18 years or had unavailable data. Female subjects constituted 63% of the sample (n = 555). Total hemoglobin values (Hb) and total white blood cell counts (WBC) were available for 869 and 872 respectively. The median age, characteristics of and complications documented in the study population with confidence intervals where applicable, are shown in Table 1. The SCD sub-types designated “Other” consisted of HbS/D, HbS/D or G, HbS/O Arab, Hb S/F and HbS-βThalassemia.

**Table 1:** Study population characteristics.

	All	Normal	Relative Hypertension	Hypertension
Age in years, Median (IQR)	31 (23–44)	27 (21–35)	30 (23–39)	50 (40–57)
Age group in years, n (%, 95%CI)				
18–20	122 (14, 12–16)	65 (21, 16–26)	49 (12, 9–16)	8 (5, 2–9)
21–24	146 (17, 14–19)	66 (21, 17–26)	71 (18, 14–22)	9 (5, 3–10)
25–30	154 (18, 15–20)	57 (18, 14–23)	91 (23, 19–27)	6 (4, 1–8)
31–39	180 (21, 18–23)	72 (23, 19–28)	92 (23, 19–28)	16 (10, 6–15)
40–54	184 (21, 18–24)	44 (14, 10–18)	70 (18, 14–22)	70 (42, 35–50)
55–76	89 (10, 8–12)	8 (3, 1–5)	25 (6, 4–9)	56 (34, 27–42)
Female gender, n (%,95%CI)	555 (63, 60–67)	205 (66,60–71)	227 (57, 52–62)	123 (75, 67–81)
Genotype, n (%, 95%CI)				
SS	394 (45, 42–48)	185 (59, 54–65)	172 (43, 38–48)	37 (22, 16–30)
SC	342 (39, 36–42)	62 (20, 16–25)	167 (42, 37–47)	113 (65, 61–75)
Other	139 (16, 14–18)	65 (21, 16–26)	59 (15, 11–19)	15 (9, 5–15)
Systolic blood pressure, Median (IQR)	117 (107–128)	107 (102–113)	119 (113–126)	136 (127–144)
Diastolic blood pressure, Median (IQR)	71 (63–79)	63 (60–67)	72 (68–78)	84 (77–89)
History of hypertension, n (%, 95%CI)	89 (10, 8–12)	0	0	89 (54, 46–62)
Taking blood pressure medication, n (%,95%CI)	78 (9, 7–11)	0	0	78 (48, 40–55)
Hemoglobin, Median (IQR)	9.0 (7.7–10.8)	11(8.4–13.3)	9.9 (7.5–12.3)	8.1 (5.9–10.4)
White blood count, Median (IQR)	9.9 (7.3–12.4)	8.0 (7.2–9.2)	9.4 (8–11.2)	10.5 (8.9–11.7)
SCD Complications, n (%, 95%CI)				
Renal	12 (1.4, 0–7.2)	4 (1.3, 0.4–3)	5 (1.3, 0.4–3)	3 (1.8, 0.4–5)
Avascular necrosis	65 (7, 6–9)	21 (7, 4–10)	29 (7, 5–10)	15 (9, 5–15)
Gallstones	35 (4, 3–6)	18 (6, 3–9)	13 (3, 2–6)	4 (2, 0.7–6)
Leg ulcer	56 (6, 5–8)	30 (10, 7–13)	23 (6, 4–9)	3 (2, 0.4–5)
Priapism	15 (5, 3–8)	7 (7, 3–13)	8 (5, 2–9)	0

The demographic features, SCD sub-types, and clinical characteristics of the study population are shown. The hemoglobin (expressed in g/dl) and total white cell count (expressed as n x 10^9^/L) are the average of data points obtained on the same days as blood pressures were taken. Renal complications comprised renal failure or insufficiency and proteinuria.

### Prevalence of RSH and hypertension

The prevalence of RSH in the study population using single most recent blood pressure was 45%. The prevalence of hypertension by the same method was 19%. The age, gender and phenotype-specific prevalence of both RSH and hypertension with 95% confidence intervals are shown in Table 2.

**Table 2:** Prevalence (95%CI) of elevated blood pressure (most recent BP measure), all values are in %.

	Elevated blood pressure	
	Relative hypertension	Hypertension	P value
Age group			
18–20	40.2 (31.4–49.4)	6.6 (2.9–12.5)	<0.001
21–24	48.6 (40.3–57.0)	6.2 (2.9–11.4)	
25–30	59.1 (50.9–66.9)	3.9 (1.4–8.3)	
31–39	51.1 (43.6–58.6)	8.9 (5.2–14.0)	
40–54	38.0 (31.0–45.5)	38.0 (31.0–45.5)	
55–76	28.1 (19.1–38.6)	62.9 (52.0–72.9)	
Gender			
Female	40.9 (36.8–45.1)	22.2 (18.8–25.9)	<0.001
Male	53.4 (47.8–59.0)	13.1 (9.6–17.3)	
Genotype			
SS	43.7 (38.7–48.7)	9.4 (6.7–12.7)	<0.001
SC	48.8 (43.4–54.3)	33.0 (28.1–38.3)	
Other	42.5 (34.1–51.1)	10.8 (6.2–17.2)	

Prevalence of RSH and hypertension with 95% Confidence Intervals (95%CI) stratified by age group, gender and genotype. Relative systemic hypertension and hypertension peak and nadir respectively within the same group of young adults, suggesting an at-risk group.

**Fig 1:**
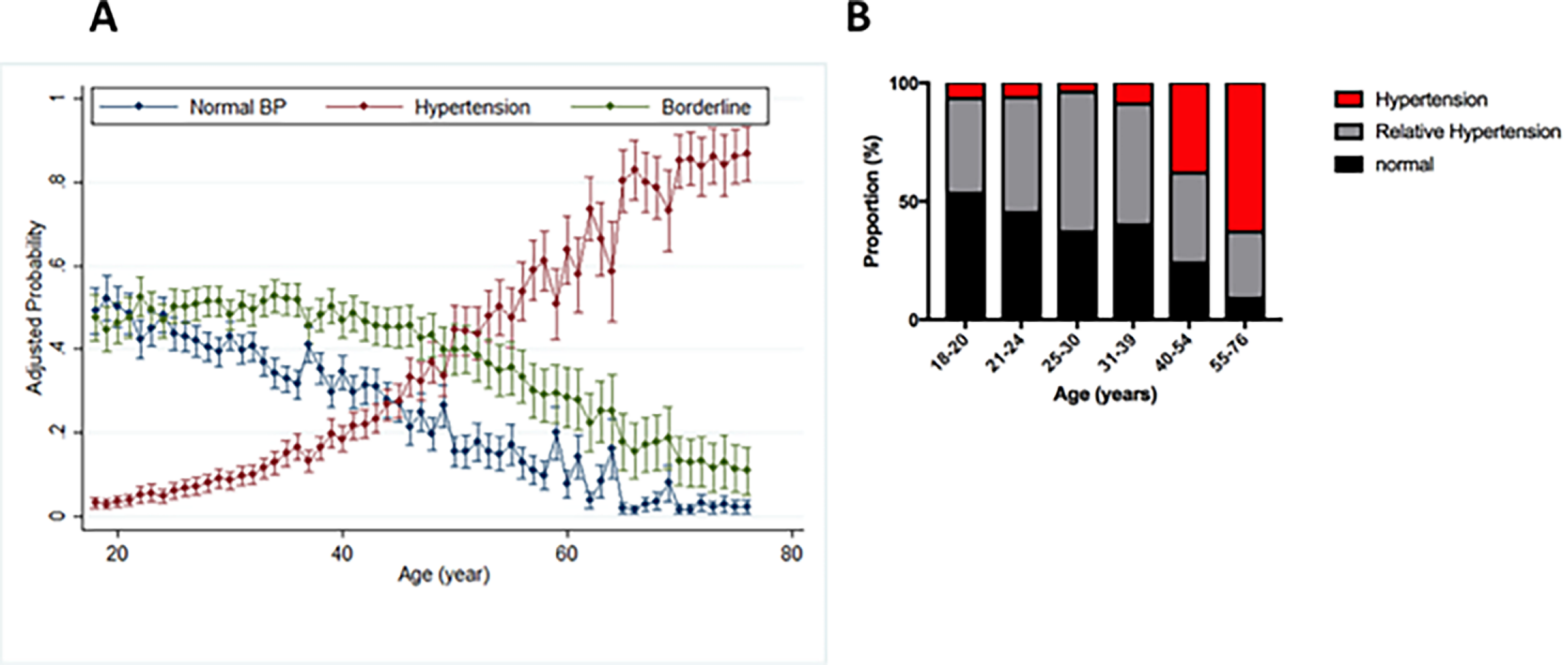
**Trends in probability of having normal BP with advancing age (A) and stratification of study population by blood pressure categories and age (B)**. Relative hypertension is denoted in (A) as borderline hypertension. Blood Pressure values were adjusted for hemoglobin and WBC count.

The probability of having normal blood pressure declined rapidly with age starting in the early twenties as relative hypertension peaked at 25–30 years. There was a marked increase in hypertension after 40 years of age (Fig 1A).

We observed a marked reduction in the proportion of young adults aged 25-30-years with hypertension as well as the peak of relative systemic hypertension in the same age group (Table 2 and Fig 1B).

### Complications of SCD related to RSH and hypertension

The most frequently documented complications in this population were avascular necrosis and leg ulcers. Initial associations of gallstones and leg ulcers with RSH did not reach statistical significance once adjusted for total hemoglobin. white blood cell count and age. We did not find any documented history of stroke in our study though this was certainly searched for. The summary of the association of complication with each blood pressure category is shown in Table 3.

**Table 3:** Percentage of different complications by blood pressure category.

	Normal	Relative Hypertension	Hypertension	Crude P value	Adjusted P value*
Renal insufficiency or proteinuria	1.3%	1.3%	1.8%	0.7	0.3
Avascular necrosis	6.7%	7.3%	9.1%	0.4	0.5
Gallstone	5.8%	3.3%	2.4%	0.05	0.08
Leg ulcer	9.6%	5.8%	1.8%	0.001	0.036
Priapism	6.5%	4.7%	0.0	0.1	0.6

* Adjusted for hemoglobin, white blood cell count and age.

No complications showed significant association with relative systemic hypertension after adjusting for hemoglobin, white blood cell count and age.

In patients who had multiple blood pressure measures with a one-year period, there was an increase of 1.4 (P = 0.005) in systolic blood pressure and an increase of 0.8 (P = 0.050) in diastolic blood pressure from the first to last measurement. None of demographic or clinical factors predicted the magnitude of time dependent changes in systolic blood pressure. Time dependent increase of diastolic blood pressure was more significant in male (P = 0.016) and younger (P = 0.026) patients.

**Fig 2:**
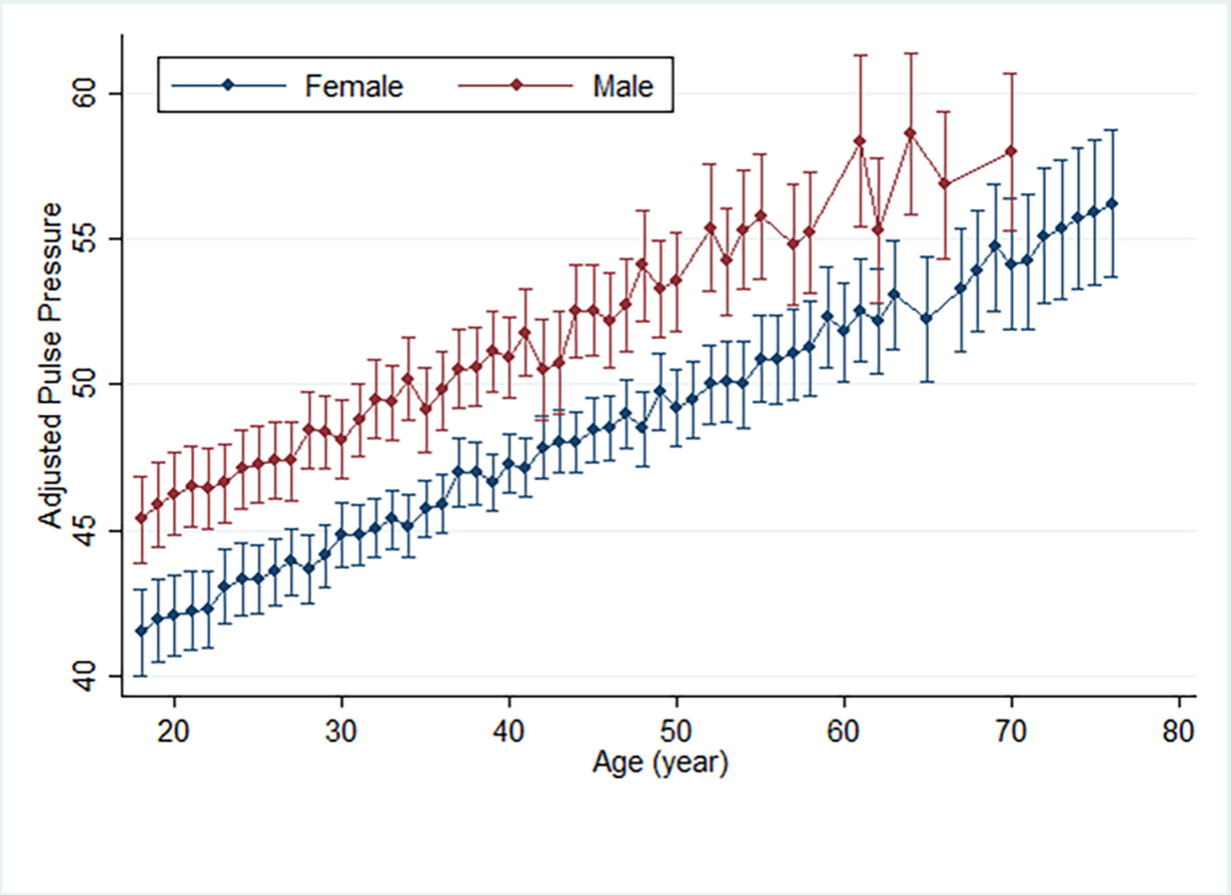
Mean pulse pressure distribution by gender and age, adjusted for hemoglobin. The median (IQR) for pulse pressure was 45 mmHg (39–55) and in linear regression analysis pulse pressure was associated with total hemoglobin (beta = -0.4 per mg/dl, P = 0.031), age (beta = 0.3 per year, P < 0.001) and male gender (beta = 4.4, P < 0.001) (Fig 2).

Pulse pressure is a marker of endothelial dysfunction and a predictor of all-cause, cardiovascular and coronary mortality. We found a higher pulse pressure in males of all ages compared to females within our study population. Pulse pressure increased with age (Fig 2).

### Repeat analysis with the average of three BP measurements

To test the reliability of our single blood pressure measurements, we repeated the analysis of our results using averaged blood pressure for subjects who had multiple measurements during a one-year period (n = 697). With this amendment, the prevalence of relative systemic hypertension and hypertension were 47% and 15%, respectively. Using this definition did not affect the predictors of relative systemic hypertension and hypertension. Also, the predictors of pulse pressure did not change using the average of three BPs.

## Discussion

### Summary of findings

Our study corroborates previous studies that describe lower blood pressure in individuals with homozygous SCD (SS) compared to those with SC. Relative systemic hypertension was present in 45% of the study population while 19% had hypertension. We demonstrate a steady increase with age in the proportion of subjects who met study-defined criteria for hypertension, with a steep rise after age 40. Most importantly, we noted a higher pulse pressure, a predictor of cardiovascular mortality, in males than females at any given age, independent of the total hemoglobin level. Complications identified in the study population are skewed towards those that are symptomatic or evident on physical exam. Young adults aged 25 to 30 years and males were identified as a particularly high-risk group for morbidity from RSH. Interestingly, we did not identify documented strokes while reviewing the charts for complications associated with SCD.

### Prevalence of relative hypertension and hypertension

Relative systemic hypertension significantly increases the risk of stroke [7] and mortality in males with SCD, those with elevated Tricuspid Regurgitant Velocity, and renal insufficiency, but is a potentially modifiable risk factor once recognized. The prevalence of RSH in our SS population is similar to the Gordeuk et al. prospective sickle cell pulmonary hypertension study which reported a 44% prevalence of RSH in 163 SS and SβThalassemia subjects [8]. Data from the Cooperative Study of SCD (CSSCD) established that blood pressures in SC are higher than in SS but lower than the general population [5]; however, as far we are aware the prevalence of RSH in SC patients has never been reported. We report a 48.8% prevalence of RSH in a large group of subjects with SC (n = 342). There was a male predominance of RSH which further highlights the risk of adverse outcomes associated with RSH in males and is likely related to gender-related differences in SCD disease biology. Interestingly, the prevalence of RSH peaked in patients > 20 years, a sub-group that other studies have identified to be at higher risk of early death [10, 11]. After this peak, prevalence of RSH declined, as the prevalence of hypertension rose. We observed a paucity of 25 to 30-year old study subjects with hypertension, possibly suggesting a disproportionately high rate of mortality or loss to clinical follow-up. This sub-group needs to be further studied with regards to causes of death, health care utilization patterns and access to comprehensive care to clarify this. It is evident that RSH is widely prevalent in this adult SCD population; we anticipate that increased awareness of SCD-specific blood pressure norms among health providers, identification of those with RSH and targeting of identified high-risk sub-groups with novel therapeutics will significantly reduce mortality.

Hypertension is reportedly uncommon in SCD. Previously published studies of hypertension in SCD report prevalence ranging from 3.2 to 10% [4, 8]. A medical history of Hypertension was the most common documented co-morbidity in our study (10%). We found a 19% prevalence of hypertension based on our study definition of the same. Data from the WHO non-communicable disease country profiles publication[12] reports a 27.3% prevalence of hypertension in the Ghanaian population, while the WHO SAGE study reports 41% prevalence of hypertension in the older population of Ghana with median age of 43 years [13]. The difference in prevalence of hypertension between our study population and that of Johnson and Giorgio is likely due to the higher numbers of elderly and SC individuals in our study (Table 1). They found only 3 of 187 subjects were aged > 54 years, so these were excluded from analysis of BP [4].The prevalence of hypertension in both our SS and “Other” sub-populations are comparable to findings from the prospective SCD pulmonary hypertension screening cohort [8].

The lack of expected age-related increase in BP has been documented in SCD, except for males in whom DBP increased slightly [5]. In contrast, more contemporary data shows no difference between BP in SCD compared to controls, and demonstrates increasing BP with age in a cohort of older SCD patients with higher BMI than previous cohorts such as CSSCD [14]. Our findings agree with Desai et al.; we noted increasing BP with age, which rose sharply over age 40 years after adjusting for hemoglobin. Furthermore, in individuals who had multiple BP measurements within a one-year period, there was an increase in systolic and diastolic BP from the first to last measurement as detailed in the results section.

We attribute these findings to the older age, SC and possibly to SCD-related organ damage. The proximity of BP in SC to that of the general population [5, 8, 15] may partly account for the high prevalence of hypertension in our study population. It is important that hypertension in SCD be diagnosed earlier and treated, given its strong association with increased mortality [16, 17]. We submit that treating hypertension in SCD may reduce mortality; though the choice of the most ideal drug for targeting the underlying cause remains in question as the pathogenesis of hypertension may differ with SCD sub-type.

The SCD-associated complications encountered in our study are listed in Table 3. Most of the renal complications were documented as renal failure, nephropathy or proteinuria. Neither leg ulcers nor gallstones had a significant association with RSH after adjusting for total hemoglobin and age. Systolic BP is a strong predictor of renal dysfunction [18]; however, we found fewer than expected instances of renal insufficiency and attribute this mostly to under-documentation of complications. For instance, only two subjects had a documented serum creatinine and pulmonary hypertension was recorded in one chart. The recent initiation of a nephrology clinic to improve patients’ access to specialty services and ongoing patient education will enhance the diagnosis of latent renal insufficiency that may exist in this population. The types of complications documented are skewed toward those associated with visible signs or physical complaints, such as leg ulcers and avascular necrosis. Relative systemic hypertension is associated with stroke. Akingbola et al, compared two cohorts of children and adults with SCD from University of Illinois (UIC), Chicago, USA and University of Ibadan, Ibadan, Nigeria. They reported a significantly higher percentage of adults aged > 18 years with a history of stroke in the UIC cohort than the Ibadan cohort (24% vs 2%, p< 0.0001)[19]. They associated the significantly higher history of stroke in the UIC cohort with higher BMI and blood pressure. One possible reason for no documented strokes in the charts of the study population is selection bias. A specialized stroke unit established to optimize the care of individuals with stroke began its operations in 2014 at the Korle-Bu Teaching Hospital, Ghana’s largest tertiary hospital and referral center, which is also the geographic location of GICG. A change in patients’ pattern of seeking medical care, death or loss to follow-up at GICG is the most likely explanation for not finding stroke documented in the charts reviewed, though it is not possible to ascertain this due to the retrospective nature of our study. It is plausible that adults with SCD who developed stroke were transported to the tertiary center and stroke unit and then continued their care with the neurologists and internists there, not necessarily returning to GICG for sickle cell- related medical care. The other possibility is that some SCD patients died after having a stroke as there is a high mortality associated with hemorrhagic stroke which is the predominant form of stroke in young adults with SCD aged 20–29 years[20].

### Systemic vascular function and pulse pressure in sickle cell disease

Interestingly, our results show elevated pulse pressure in males more than females at all ages. This is likely an important part of reason behind the greater mortality noted in males with SCD. Elevated pulse pressure (PP) has emerged as a strong predictor of all-cause and cardiovascular and coronary mortality and is considered an indirect measure of proximal aortic stiffness [21]. In SCD, high pulse pressure is associated with markers of hemolysis [18] and may contribute to the sudden deaths seen in SCD, [1]. A possible explanation for the higher pulse pressure noted in males in our study is gender-based differential responsiveness of the vasculature to nitric oxide [22]. Pulse pressure, linked in so many ways to SCD vasculopathy and risk of cardiovascular death, could prove to be a valuable marker of the adequacy of a response to the ideal therapeutic agent for RSH in clinical trials.

### Strengths and limitations

The strengths of the study are in the large number of subjects, providing a representative sample of both SS and SC, which make our findings more generalizable. This will facilitate subject enrollment or clinical trials and expedite the translation of novel disease-modifying therapies for SCD worldwide.

The limitations of this study are in its retrospective nature and the limited resources at the site of study, which limited our ability to obtain more specific information on Hb types and complications in steady state. Our sample may have inherent selection bias of individuals that seek care more frequently. Some of the blood pressure measurements were done during patient visits for pain or acute illness, situations that can temporarily elevate or lower blood pressure. We did not stratify our study population by the reason for their visit. To reduce this effect, we performed a repeat analysis of a subset of the charts reviewed using 3 blood pressure values taken on different days and if a blood pressure reading was repeated on the same day (which is not a typical process as observed from chart reviews), we used the second value for that day, presuming the health provider perceived the first value to be abnormal. This introduces a limitation itself but acknowledges that the patients’ clinical complaint or reason for visiting the GICG could modify their baseline blood pressure.

## Conclusions

Relative systemic hypertension is prevalent in the adult SCD population in Ghana. Young adults aged 25 to 30 years, who have the highest prevalence of RSH, and males with high pulse pressures above a threshold previously associated with increased mortality have emerged as two high-risk sub-groups within the population requiring intervention.
